# Antiproliferative effect of alpinetin in BxPC-3 pancreatic cancer cells

**DOI:** 10.3892/ijmm.2012.884

**Published:** 2012-01-11

**Authors:** JIAN DU, BO TANG, JINGWEN WANG, HONGTAO SUI, XUELI JIN, LIMING WANG, ZHONGYU WANG

**Affiliations:** 1 Department of General Surgery, The First Affiliated Hospital of Dalian Medical University, Dalian 116011; 2 Department of General Surgery, The Second Affiliated Hospital of Dalian Medical University, Dalian 116027, P.R. China; 3 Department of Cell and Molecular Biology, Uppsala University, Uppsala, Sweden

**Keywords:** alpinetin, pancreatic cancer, proliferation, apoptosis, caspases, cell cycle

## Abstract

Alpinetin is a novel plant flavonoid derived from *Alpinia katsumadai* Hayata, found to possess strong anticancer effects. However, the antitumor effect of alpinetin on pancreatic cancer cells and the detailed mechanism remain unclear. The aim of this study was to investigate alpinetin's beneficial effect on pancreatic cancer and the possible molecular mechanism involved. Pancreatic cancer cell lines were treated with alpinetin at various doses and for different times, and the effect of alpinetin on cell growth inhibition, apoptosis and the cell cycle was determined. The expression of Bcl-2, Bcl-xL, XIAP and Bax, the activity of caspases and the levels of cytochrome c released were measured. The results showed that alpinetin inhibited the viability of three pancreatic cancer cell lines and induced apoptosis of BxPC-3 cells in a dose- and time-dependent manner. This was accompanied by regulation of the expression of Bcl-2, Bcl-xL, Bax and XIAP. Furthermore, alpinetin treatment led to the release of cytochrome c and activation of caspases-3, −8 and −9 proteins. Taken together, our studies indicate that alpinetin inhibited the proliferation of pancreatic cancer cells possibly through the regulation of the Bcl-2 family and XIAP expression, release of cytochrome c and the activation of caspases. Alpinetin may serve as a potential agent for the development of pancreatic cancer cell therapies.

## Introduction

Pancreatic cancer is one of the most common malignant tumors in the world. In addition, it is the fourth most common cause of cancer-related deaths with an overall 5-year survival rate of <2% (1). Surgical resection represents the main treatment, but only a minority (10–20%) of patients are eligible for surgical treatment due to difficulties in early diagnosis (2–4). The results of chemotherapy and radiotherapy are not satisfactory because some tumor cells are capable of evading drug or radiation-induced cell death (5,6).

### Alpinia katsumadai

Hayata, as a traditional medicine with low toxicity, has notable of antitumor and anti-oxidative effects (7,8). Alpinetin, 7-hydroxy-5-methoxyflavanone (molecular formula C_16_H_14_O_4_, molecular weight 270.28), found in *Alpinia katsumadai* Hayata, is a novel plant-derived flavonoid and is believed to be the major active ingredient of *Alpinia katsumadai* Hayata (9,10). Previous studies demonstrated blockade of the proliferation of the human tumor cells by alpinetin, indicating the potential anticancer properties of this compound. The anticancer capability of alpinetin has also been confirmed in the treatment of breast cancer, hepatoma, leukemia, carcinoma of the colon and pulmonary cancer (11–13). However, the antitumor effect of alpinetin on pancreatic cancer cells and the detailed mechanisms involved in it remain largely unknown.

It has been suggested that pancreatic cancer cells have protective mechanisms against the mitochondrial pathway of apoptosis through overexpression of Bcl-family proteins or XIAP to block activation of caspases (14). Previous studies also proved that Bcl-2 and XIAP protein are two important targets for antitumor medicines (15,16).

The aim of this study was to investigate the anticancer effect and the possible mechanisms of alpinetin on pancreatic cancer cells. BxPC-3 is an extremely metastatic human pancreatic cancer cell line, chosen for detailed study. We found that alpinetin can induce human pancreatic cancer cells apoptosis, possibly through regulation of the Bcl-2 family and XIAP expression and of the release of cytochrome c.

## Materials and methods

### Cell culture, antibodies and reagents

The BxPC-3, PANC-1 and AsPC-1 human pancreatic cancer cell lines were purchased from the American Type Culture Collection (ATCC). Cells were cultured in RPMI-1640 medium with 10% fetal bovine serum (FBS) and maintained at 37°C in 5% CO_2_. Alpinetin (≥98% purity) was obtained from the National Institute for Food and Drug Control (Beijing, China). Bcl-2, Bcl-xL, Bax, XIAP and GAPDH antibodies were from Cell Signaling Technology, Inc. (USA). Propidium iodide (PI) and Annexin V- fluorescein isothiocyanate (FITC) were from Sigma (USA). Hoechst 33342 was from Beyotime (China). Fluorogenic caspase substrates Ac-DEVD-AMC (acetyl-Asp-Glu-Val-Asp-aminomethylcoumarin), Ac-IETD-AMC (acetyl-Ile-Glu-Thr-Asp-aminomethylcoumarin) and Ac-LEHD-AMC (acetyl-Leu-Glu-His-Asp-aminomethylcoumarin) were from Alexis Biochemicals (San Diego, CA).

### Cell proliferation assay

The effect of alpinetin on cell proliferation was detected using methyl-thiazolyl-terazolium (MTT) (Sigma) assay. Cells growing in logarithmic phase were seeded in the 96-well plate and then treated with alpinetin. Twenty microliters of MTT (0.5 mg/ml) was added to each well followed by incubation at 37°C for 4 h to allow the yellow dye to be transformed into blue crystals. The medium was removed and 200 μl of dimethyl sulfoxide (DMSO) (Sigma) was added to each well to dissolve the dark blue crystals. Finally, the optical density was measured with a microtiter plate reader at 570 nm. Six replicates were prepared for each condition.

### Hoechst 33342 nuclear staining

Pancreatic cancer cells were plated in 6-well plates with poly-lysine-coated coverslips and cultured for 24 h. Then the cells were treated with or without alpinetin for 24 h. The untreated and treated cells were washed twice with PBS and incubated with 8 μg/ml Hoechst 33342 (Sigma) at 37°C for 20 min, and fluorescent images were obtained using a fluorescence microscope (Leica Microsystems, Germany).

### Annexin V-FITC/PI double-labeled detection of apoptosis

The protocol was based on the use of Annexin V-FITC and PI staining according to the manufacturer's instructions. The results were analyzed by flow cytometry to differentiate the types of cell death. Cells that were Annexin V-FITC-positive and PI-negative were classified as apoptotic or early-stage apoptotic cells. Briefly, cells were digested with 0.25% trypsin and washed three times with PBS. Unfixed cells were stained by adding the Annexin V-FITC reaction mixture (10 μl Annexin V-FITC, 5 μl propidium iodide) and incubated at room temperature for 15 min in the dark. The stained cells were subjected to flow cytometric analysis with a FACSCalibur (Becton-Dickinson, USA).

### Caspase activity assay

The activities of caspases-3, −8 and-9 were checked according to the procedures of Köhler *et al* with minor modifications (17). Briefly, the cells were collected and suspended in extraction buffer (50 mmol/l Tris-HCl, pH 7.4; 10 mmol/l EGTA; 1 mmol/l EDTA; 10 mmol/l DTT; 1% (v/v) Triton X-100). Subsequently, the supernatants were stained by 20 mmol/l fluorogenic peptide substrates, Ac-DEVD-AMC (caspase-3), Ac-IETD-AMC (caspase-8) and Ac-LEHD-AMC (caspase-9) for 30 min at 37°C. Fluorescence was checked on a Perkin-Elmer LS-50B spectrofluorimeter, setting excitation at 380 nm and emission at 460 nm. Changes in caspase activities were determined by comparing the levels of the alpinetin-treated cells with the controls.

### Mitochondria separation, protein extraction and western blot analysis

Mitochondria in BxPC-3 cells were separated and extraction was performed according to the procedures described in our previous study (18). Briefly, cells were washed once with ice-cold PBS containing 100 mM sodium orthovanadate and solubilized in lysis buffer (50 mM Tris-HCl, 137 mM NaCl, 10% glycerol, 100 mM sodium orthovanadate, 1 mM phenylmethylsulphonyl fluoride (PMSF), 10 mg/ml aprotinin, 10 mg/ml leupeptin, 1% Nonidet P-40; pH 7.4). After centrifugation at 12,000 × g for 20 min, the supernatant was collected. Cells were dissolved in sample buffer containing 65 mM Tris-HCl (pH 6.8), 3% SDS, 10% glycerol and 6 M urea. After determination of the protein concentration (BCA kit; Pierce, Rockford, IL, USA), β-mercaptoethanol and bromophenol blue were added to the sample buffer for electrophoresis. The protein was separated by 12% SDS-polyacrylamide gel electrophoresis (PAGE) and transblotted to polyvinylidene difluoride membranes (Bio-Rad, Hercules, CA, USA). The blots were incubated at 4°C overnight with antibodies, and the resulting bands were detected using enhanced chemiluminescence. Intensities of the bands were quantified using an image-analysis system.

### Analysis of the cell cycle by flow cytometry

Cells in the logarithmic phase were seeded in 6-well plates and then treated with alpinetin. After collection by centrifugation and washing twice with PBS, the cell pellets were suspended in 1 ml ice-cold 70% ethanol at 4°C. After 1 h, the fixed cells were spun by centrifugation and the pellets were washed with PBS. After resuspension with 1 ml PI integration staining solution, the cells were incubated with RNase A (10 mg/l), PI (50 mg/l), 1% Triton X-100 and sodium citrate (1 g/l) shaken for 30 min at 37°C in the dark. The stained cells were analyzed using a FACSCalibur flow cytometer (Becton-Dickinson).

### Statistical analysis

All data are expressed as the mean ± standard deviation (SD). One-way ANOVA and the unpaired Student's t-test was used to test for differences between the two groups. A P-value <0.05 was considered statistically significant.

## Results

### Alpinetin inhibits the growth of pancreatic cancer cells

**Figure 1:**
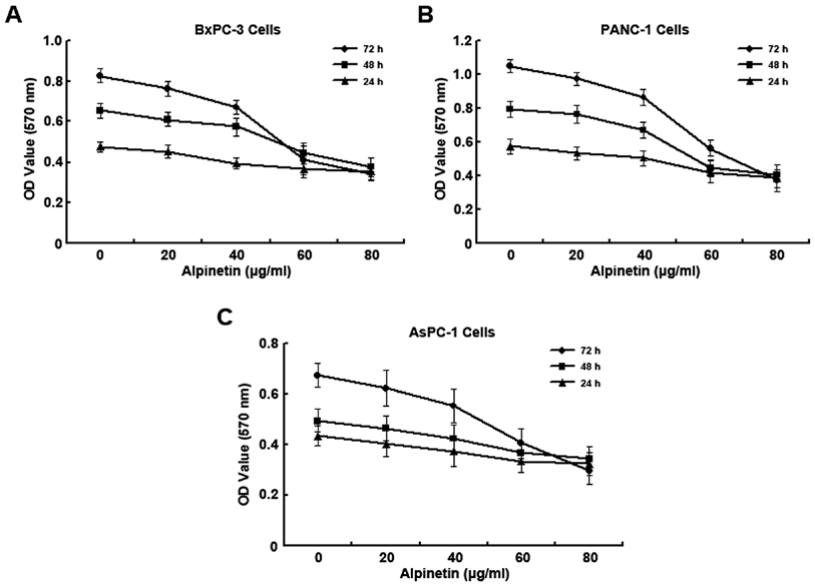
Inhibitory effect of alpinetin on the growth of human pancreatic cancer cell lines. BxPC-3, PANC-1 and AsPC-1 cells were treated with different concentrations of alpinetin (0, 20, 40, 60 and 80 μg/ml) for 24, 48 and 72 h respectively. Cell proliferation was then determined by the MTT assay. The results are representative of six independent experiments.

To investigate the effect of alpinetin on proliferation and viability of pancreatic cancer cells, BxPC-3, PANC-1 and AsPC-1 pancreatic cancer cells were treated with alpinetin at different doses (0, 20, 40, 60, 80 μg/ml) for 24, 48 or 72 h, and then an MTT assay was performed to determine cell viability. The results suggested that the viability of three alpinetin-treated pancreatic cancer cell lines was greatly decreased with increased alpinetin dose and treatment time (Fig. 1). At concentrations ranging from 0 to 40 μg/ml, slight growth inhibition effects on the pancreatic cancer cells was observed. The concentration of alpinetin needed to significantly inhibit cell growth of BxPC-3 and PANC-1, AsPC-1 cells was 60 μg/ml. The effect of inhibition in pancreatic cancer cells increased proportionately when treated with at a range from 20 to 80 μg/ml alpinetin for 48 h. BxPC-3 cells are an extremely metastatic human pancreatic cancer cell line compared to the other two cell lines examined herein. We thus chose to treat BxPC-3 cells with 20 to 80 μg/ml alpinetin for 48 h to further investigate its detailed mechanism of action.

### Alpinetin induces BxPC-3 pancreatic cancer cells apoptosis

**Figure 2:**
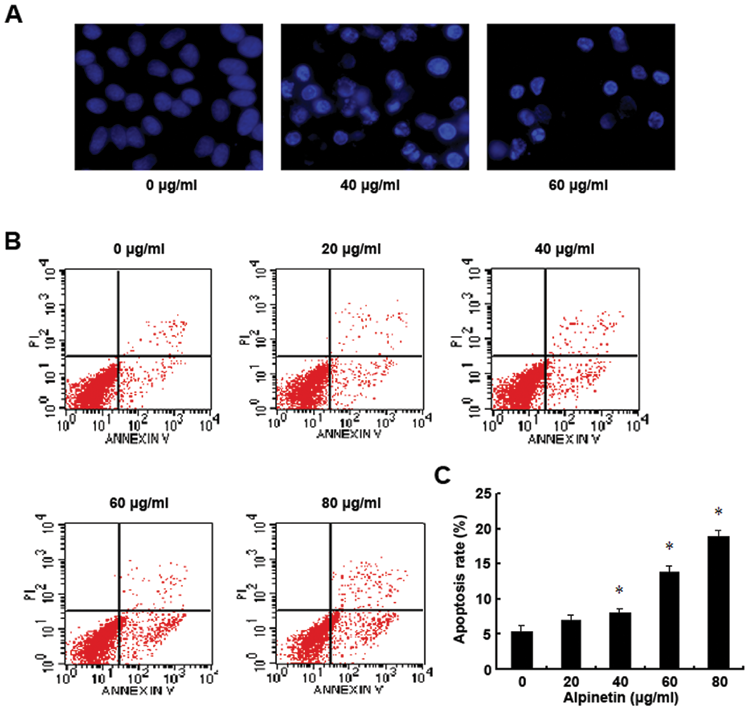
Alpinetin induces BxPC-3 pancreatic cancer cells apoptosis. BxPC-3 cells induced by different concentrations of alpinetin for 48 h were identified using (A) Hoechst 33342 nuclear staining and analyzed by (B) Annexin V-FITC/PI double stained flow cytometry. (C) The apoptosis rate (%) in BxPC-3 pancreatic cancer cells are shown by a histogram (^*^P<0.05). The data are representative of three independent experiments.

To explore whether the inhibitory effects of alpinetin on pancreatic cancer cells was attributed to the induction of apoptosis, BxPC-3 cells were treated with alpinetin at different doses for 48 h. Then the alpinetin-treated cells were identified using Hoechst 33342 nuclear staining and analyzed by Annexin V-FITC/PI double staining flow cytometry (Fig. 2). As shown in our results, the condensed chromatin of apoptotic cells in the 40 and 60 μg/ml alpinetin-treated groups was significantly brighter than the chromatin of normal cells in the control group (Fig. 2A). Furthermore, alpinetin-treated BxPC-3 cells had notably higher apoptosis rates which were dose-dependent compared to the control group (Fig. 2B and C). These results indicate that alpinetin dose-dependently induced cell apoptosis to inhibit proliferation of pancreatic cancer cells.

### Effect of alpinetin on caspase activation in BxPC-3 cells

**Figure 3:**
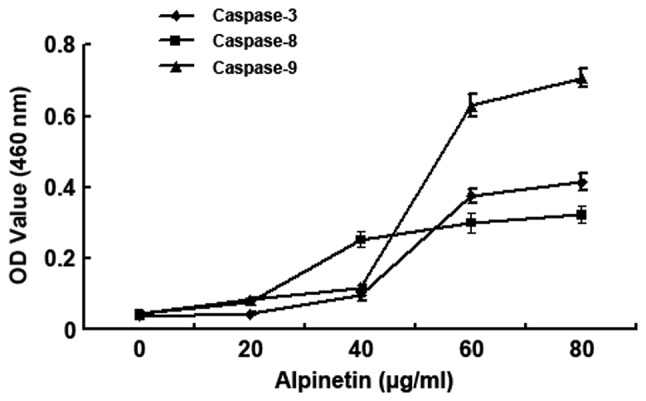
Effect of alpinetin on the caspase −3, −8 and −9 activation in BxPC-3 pancreatic cancer cells. After 48 h incubation with different concentrations of alpinetin, the activation of caspases in BxPC-3 cells was assessed by a spectrofluorimeter and illustrated by a line chart. The data are representative of three independent experiments.

To detect the apoptotic pathway induced by alpinetin, BxPC-3 cells were treated with different concentrations of alpinetin for 48 h, and then the activities of caspases-3, −8 and −9 were determined by the fluorogenic peptide substrates Ac-IETD, Ac-IETD-AMC and Ac-LEHD-AMC, respectively (Fig. 3). Our data show that the caspase-8 and −9 activities were significantly increased in BxPC-3 cells treated with 60 μg/ml alpinetin. As a downstream effector of caspase-8 and −9, the activity of caspase-3 was found to be 3- to 4-fold higher after treatment with 40 μg/ml alpinetin and reached its peak at 60 μg/ml in alpinetin-treated BxPC-3 cells. Our data suggest that alpinetin could induce caspase activation and caspase-dependent apoptosis in BxPC-3 cells.

### Alpinetin regulates the expression of the Bcl-2 family members and of XIAP and the release of cytochrome c in BxPC-3 cells

**Figure 4:**
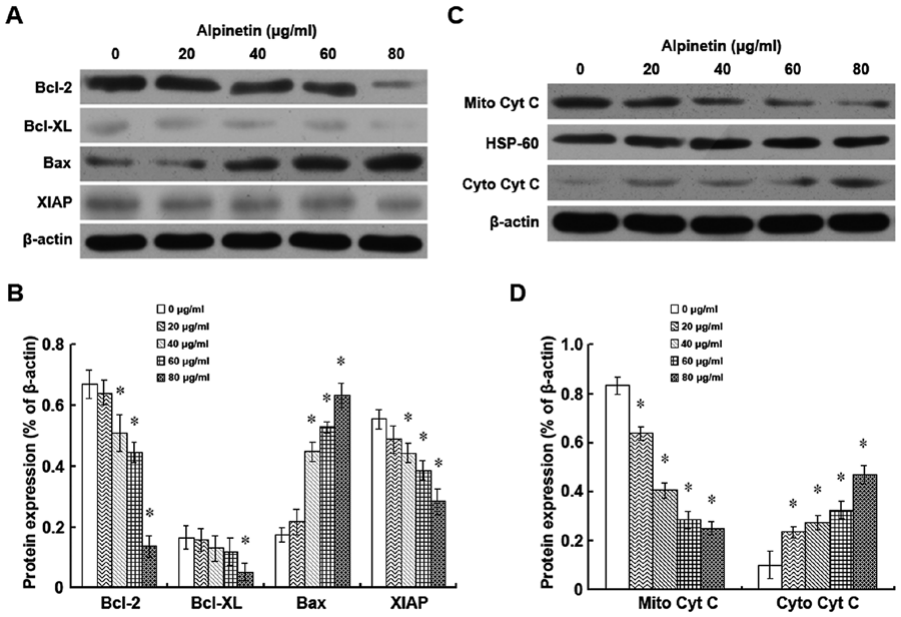
The effect of alpinetin on the protein expression of Bcl-2 family members and XIAP and on the release of mitochondrial cytochrome c. BxPC-3 cells were treated with different concentrations (0, 20, 40, 60 and 80 μg/ml) of Alpinetin for 48 h. (A) The expression of Bcl-2, Bcl-xL, Bax and XIAP were determined by western blot analysis. (C) The expression of cytochrome c in mitochondria and cytoplasm were also examined by western blot analysis. (B and D) The western blot results were further analyzed using Gel-Pro Analyzer 4.0 software (^*^P<0.05). Results are representative of three independent experiments.

To further study the mechanism involved in alpinetin-induced cell growth inhibition and apoptosis, the changes in the expression of the Bcl-2 family members and XIAP and in the release of cytochrome c were assessed by western blot analysis following treatment with alpinetin for 48 h (Fig. 4). Our data showed that the expression of the anti-apoptotic proteins Bcl-2, Bcl-xL and XIAP in BxPC-3 cells were downregulated by alpinetin in a dose-dependent manner. Conversely, the expression of the pro-apoptotic protein Bax was upregulated in alpinetin-treated BxPC-3 cells (Fig. 4A and B). Also, the expression of cytochrome c in mitochondria was remarkably decreased while a significant increase of cytochrome c protein levels in the cytoplasm was observed after alpinetin treatment (Fig. 4C and D). All these results indicate that alpinetin could activate the mitochondrial apoptotic pathway which could be responsible for its ability to suppress proliferation of BxPC-3 cells.

### Effects of alpinetin on cell cycle phase distribution

**Figure 5:**
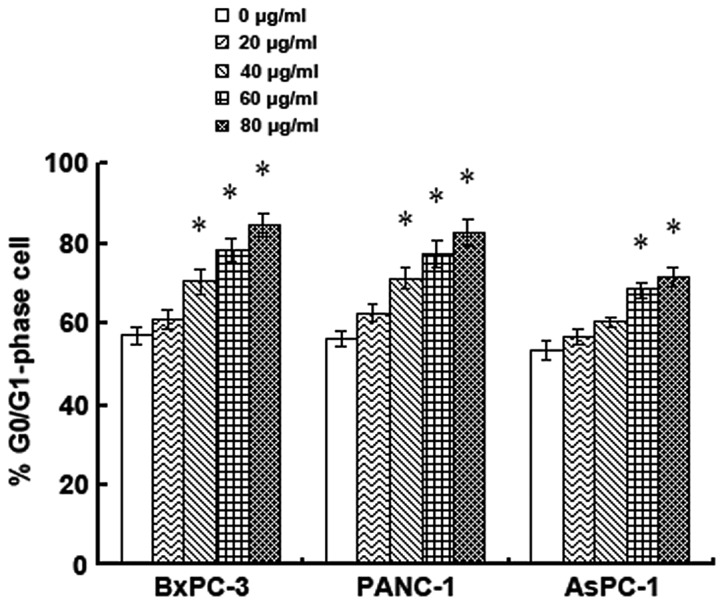
G0/G1-phase arrest induced by alpinetin in pancreatic cell lines. BxPC-3, PANC-1, AsPC-1 cells were treated with alpinetin (0, 20, 40, 60 and 80 μg/ml) for 24 h, and then the distribution of cell cycle was determined by flow cytometry. Cell cycle progression in alpinetin-treated three pancreatic cancer cell lines was assessed using flow cytometry. The percentages of G0/G1 phase cell in different groups are shown by columns (^*^P<0.05). These results shown represent means ± SD from three independent experiments performed in triplicate.

To investigate whether alpinetin could regulate cell cycle phase distribution in human pancreatic cancer cells, cell cycle progression in 3 alpinetin-treated pancreatic cancer cell lines was assessed using flow cytometry. The percentage of different treatment groups in the G0/G1 phase are shown by histograms (Fig. 5). As shown in our results, an arrest in the G0/G1 phase was observed after treatment with 40 μg/ml alpinetin for 48 h in BxPC-3 cells. Meanwhile, the percentage of PANC-1 cells in the G0/G1 phase was also higher in the 40 μg/ml alpinetin-treated group than in the untreated cells. In AsPC-1 cells, alpinetin concentrations of 60 μg/ml and higher caused an arrest in the G0/G1 phase. Our data implies that the anti-proliferation effect induced by alpinetin also possibly occurs through inducing G0/G1 phase arrest.

## Discussion

Our present study shows that alpinetin, the extract of a traditional Chinese herb, is able to remarkably inhibit the growth and viability of three human pancreatic cancer cells. Furthermore, it has been indicated that alpinetin induces BxPC-3 cells apoptosis through upregulating pro-apoptotic Bax expression and downregulating anti-apoptotic Bcl-2, Bcl-xL and XIAP expression and results in the release of cytochrome c to the cytoplasm and the activation of caspases-3, −8, −9. Moreover, alpinetin can also induce G0/G1 phase arrest in human pancreatic cancer cells. These results suggest that the effect of alpinetin on suppressing the proliferation and viability of pancreatic cancer cells may be mediated by regulating Bcl-2 family and XIAP expression, releasing of cytochrome c and activation of caspases.

It has been revealed that alpinetin exerts anti-proliferative activity against various types of tumors such as hepatoma, breast carcinoma and leukemia (11). Keeping in line with these observations, we confirmed that alpinetin showed strong antitumor activity in three human pancreatic cancer cell lines. However, less is known regarding defined signaling pathways involved in these processes. In this study, Hoechst 33342 nuclear staining and FACS analysis revealed that treatment of BxPC-3 pancreatic cancer cells with alpinetin for 48 h increased condensed chromatin and Annexin V-positive and PI-negative populations in a dose-dependent manner, which suggested that part of the alpinetin induced suppression was mediated through the induction of apoptosis rather than the necrosis. The observation that alpinetin treatment induced cell cycle arrest at the G0/G1 phase further supports that alpinetin-induced BxPC-3 pancreatic cancer cell death occurs through regulation of apoptosis.

Apoptosis is essential for the fundamental antitumor mechanism which prevents tumorigenesis of normal cells (6). There are two alternative intracellular pathways, the intrinsic or mitochondrial pathway and the extrinsic or death-receptor pathway to initiate apoptosis (19). The Bcl-2 family, which is associated with the intrinsic apoptosis pathway, plays a crucial role in the control of apoptosis by the activation of caspases or by regulating the release of cytochrome c (20–22). This family includes numerous proteins with homologous amino acid sequences and some of them have the ability to promote apoptosis, such as Bax, while others have anti-apoptotic effects, like Bcl-2, Bcl-xL and XIAP. The relative effect of these pro- and anti-apoptotic Bcl-2 family members regulates the death signaling pathways in cells (23,24). Many studies have shown that pancreatic cancer cells, like other tumor cells, could alter the balance of pro- and anti-apoptotic proteins, therefore attenuating apoptosis processes induced by anticancer drugs (25,26). Previous studies also indicated that pancreatic cancer cells suppress the mitochondrial apoptotic pathway by overexpression of Bcl-2, Bcl-xL or XIAP to block the activation of caspases (14). In this study, to confirm the possible mechanism involved in alpinetin-induced apoptosis, the expression of the anti-apoptotic proteins Bcl-2, Bcl-xL and XIAP as well as of the pro-apoptotic protein Bax were examined. We found that the protein expression of Bcl-2, Bcl-xL and XIAP were significantly suppressed in alpinetin-treated BxPC-3 cells. Meanwhile, the expression of Bax was remarkably upregulated after treatment with alpinetin. The data were consistent with the previous reports that overexpression of anti-apoptotic proteins such as Bcl-2 and Bcl-xL repress the function of Bax. Conversely, upregulation of Bax expression promotes cell death (27,28).

Caspases, a family of cysteine proteases which cleave protein substrates after their Asp residues and play important roles in the extrinsic and intrinsic apoptotic response. In the mitochondrial-dependent apoptotic pathway, Bcl-2 reduces the mitochondrial permeability and prevents cytochrome c release to the cytoplasm while Bax has the converse effect. Cytochrome c released from the mitochondria can activate the initiator caspase-9 and the effector caspase-3, which is an important step in the apoptotic pathway (29,30). Our present study showed that the activations of caspases-3, −8 and −9 in alpinetin-treated BxPC-3 cells were enhanced in a dose-dependent manner. Moreover, the release of cytochrome c from the mitochondria to the cytoplasm was also relatively increased. Meanwhile, activated caspase-8, as an upstream signaling molecule in caspase cascades, could induce caspase-9 activation. These findings are similar to a study of apoptosis in breast cancer cells (31).

In conclusion, we have demonstrated that alpinetin inhibit the proliferation and viability of pancreatic cancer cells in a dose- and time-dependent manner through regulating Bcl-2 family and XIAP expression, releasing of cytochrome c and activation of caspases. Alpinetin may be a potential traditional Chinese medicine for the future development of pancreatic cancer therapy.
